# Influenza and Memory T Cells: How to Awake the Force

**DOI:** 10.3390/vaccines4040033

**Published:** 2016-10-13

**Authors:** Jan Spitaels, Kenny Roose, Xavier Saelens

**Affiliations:** 1 Medical Biotechnology Center, VIB, Ghent 9000, Belgium; jan.spitaels@vib-ugent.be (J.S.); kenny.roose@vib-ugent.be (K.R.); 2 Department of Biomedical Molecular Biology, Ghent University, Ghent 9000, Belgium

**Keywords:** influenza, vaccines, cellular immunity, memory T cells

## Abstract

Annual influenza vaccination is an effective way to prevent human influenza. Current vaccines are mainly focused on eliciting a strain-matched humoral immune response, requiring yearly updates, and do not provide protection for all vaccinated individuals. The past few years, the importance of cellular immunity, and especially memory T cells, in long-lived protection against influenza virus has become clear. To overcome the shortcomings of current influenza vaccines, eliciting both humoral and cellular immunity is imperative. Today, several new vaccines such as infection-permissive and recombinant T cell inducing vaccines, are being developed and show promising results. These vaccines will allow us to stay several steps ahead of the constantly evolving influenza virus.

## 1. Introduction

Annually, human influenza virus causes three to five million cases of severe illness and about 250,000 to 500,000 deaths worldwide [1]. This, however, only represents the burden of seasonal influenza virus infections. During the last century, four influenza pandemics occurred: in 1918 (H1N1), 1957 (H2N2), 1968 (H3N2), and 1977 (H1N1) [2]. The most recently declared human influenza pandemic emerged in 2009 and was caused by a swine origin H1N1 virus [3]. It is estimated that globally over 200,000 people succumbed to this virus within the first year after its outbreak in the spring of 2009 [4].

Influenza virus infection induces a profound humoral and cellular immune response in the host. Serum IgG antibodies that are directed to influenza virus hemagglutinin (HA) and neuraminidase (NA) glycoproteins correlate with protective immunity [5,6]. Influenza viruses evade this humoral response by a process named “antigenic drift”. This phenomenon is driven by the relatively high frequency of misincorporated nucleotides into the genomes of the viral progeny by the viral RNA-dependent RNA polymerase complex [7]. Immune pressure exerted by antibodies that inhibit HA or NA function can lead to the selection of viruses from this diverse genetic pool that can escape humoral immunity. Cellular immunity on the other hand is mainly directed against more conserved internal influenza viral proteins such as the nucleoprotein (NP) [8]. Within this arm of the adaptive immune system, CD8^+^ T cells are very important for virus clearance, and are able to provide heterosubtypic immunity [9]. The importance of CD8^+^ T cell immunity in the host control of influenza virus infection is illustrated by the higher incidence of mutations in the T cell epitope regions of e.g., NP compared to the rest of the protein, which indicates that these epitopes are under selective pressure [10,11].

Annual vaccination is considered the most effective way to prevent disease caused by influenza A and B virus infection [12]. In many countries this practice is recommended for all persons older than six months who do not have contraindications, and especially for elderly people, immunocompromised persons and children [13]. The virus strains that are included in these vaccines are recommended by an expert panel of the World Health Organization (WHO) that bases its predictions on influenza surveillance data (Global Influenza Surveillance and Response System).

Most of the current licensed influenza vaccines are tri-(TIV) or quadrivalent (QIV) inactivated formulations that contain 15 μg each of the hemagglutinin glycoprotein of two influenza A strains (H1N1 and H3N2) and one or two influenza B strains (Yamagata and Victoria lineage). Inactivated influenza vaccines are administered by intramuscular injection or, less frequently, by intradermal injection. Mostly, these types of vaccines are derived from viruses grown in fertilized chicken eggs. However, it is worth mentioning Flucelvax^®^, which is an FDA approved TIV that is manufactured using mammalian cell culture technology [14]. Alternatively, a live-attenuated influenza vaccine (LAIV) that is administered intranasally is available in the USA and some European countries. This vaccine is licensed for use in healthy individuals aged 2–49 years [13]. This vaccine is produced by reassortment of the selected influenza virus strain with the cold-adapted A/Ann Arbor/6/1960 vaccine strain. It only replicates efficiently at a temperature of 25 °C (in the nasal cavity), but very poorly at higher temperatures in the human body (such as the lower respiratory tract) [15]. In the United States, the effectiveness of LAIV in comparison to inactivated vaccines was quite low in subjects aged 2–17 years during influenza seasons 2013–2014 and 2015–2016, especially against influenza A/H1N1pdm09 [16,17]. Therefore, the CDC’s Advisory Committee on Immunization Practices (ACIP) decided not to recommend LAIV for use during the 2016–2017 influenza season [18]. Flublok^®^ is a third type of human influenza vaccines. This vaccine is a trivalent, seasonal influenza vaccine consisting of three full-length recombinant hemagglutinin influenza virus proteins. The vaccine is produced in an insect cell line derived from Sf9 cells of the fall armyworm, *Spodoptera frugiperda*. Each of the three HAs is expressed using a viral vector (baculovirus *Autographa californica* Nuclear Polyhedrosis Virus) that is non-pathogenic for humans.

However, these vaccines do not elicit a strong heterosubtypic immune response, since the majority of the vaccine-induced antibodies fail to cross-react with hetero(sub)typic HA and NA, and if cross-reactive T cell responses are induced, these responses are much lower than the homologous T cell response [19,20]. It was shown that there were no increases in the mean levels of influenza A virus-reactive IFN-γ^+^ T cells and NK cells in adults given either LAIV or TIV while LAIV did have a positive effect on influenza A virus-specific IFN-γ^+^ CD4^+^ and CD8^+^ T cells in children aged 5–9 years [21]. Additionally, TIV treatment had a significant effect in 6-month to 4-year-old children on the level of influenza A virus-reactive T cells; LAIV was not evaluated in this age group. This indicates that the efficacy of inducing a cellular immune response of currently used vaccines is highly dependent on age, type of vaccine, and prevaccination levels of immune reactivity to influenza A virus [21]. In young children, who are often immunologically naïve to influenza virus, inactivated vaccines may even hamper the induction of cell-mediated immunity that would be otherwise induced by natural (disease causing) infections [22]. Hence, the big challenge in influenza vaccine development remains the induction of broadly neutralizing antibodies and long-lasting heterosubtypic cellular immune responses.

## 2. Immune Response to Influenza Virus Infection

### 2.1. Innate Immunity

#### 2.1.1. Extracellular Barriers to Overcome

Before it can infect respiratory epithelial cells, the influenza virus has to cross or circumvent two main barriers. The first barrier is the mucus layer that lines the respiratory tract. This layer forms a physical barrier consisting of a mixture of cells, cellular debris and polypeptides, held together by macromolecular constituents called mucins. Mucins are a family of glycoproteins that are secreted or remain membrane associated. They are heavily glycosylated, and the terminal sialic acid residues of these glycans are linked to galactose. It has been shown that upon viral infection of the respiratory tract, the production of mucus in the epithelial surfaces of the respiratory tract increases [23,24]. To cross this mucus layer, influenza viruses rely on the enzymatic activity of NA, which cleaves off terminal sialic acids from glycans [25].

**Figure 1:**
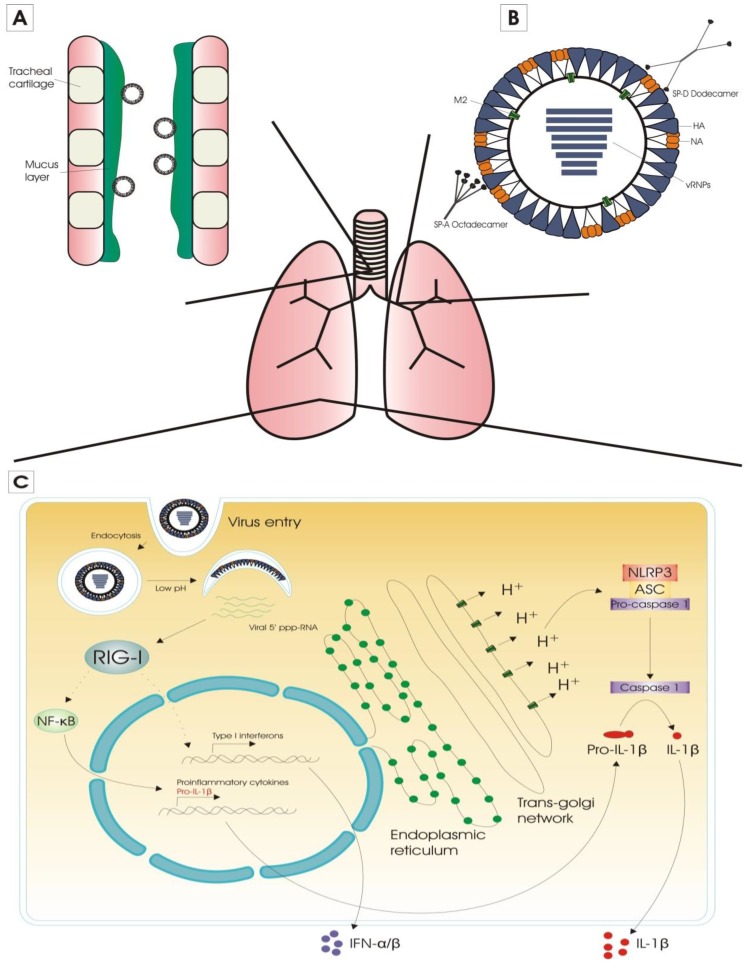
Innate immunity against influenza virus infection. (**A**) The first barrier that the influenza virus has to overcome, is the mucus layer that lines the respiratory tract. To cross this barrier, influenza viruses rely on the enzymatic activity of the neuraminidase glycoprotein; (**B**) The second barrier consists of carbohydrate-binding proteins called lectins. Surfactant proteins A (SP-A) and D (SP-D) are the main two lectins involved in anti-influenza activity. SP-A acts as a decoy receptor for influenza virus, and SP-D binds to oligosaccharides on influenza hemagglutinin (HA) and neuraminidase (NA) proteins; (**C**) Once influenza virions have reached respiratory epithelial cells they recognize sialic acid-containing host cell receptors by the HA glycoprotein. This is followed by endocytosis of the influenza virus and the virion particle ends up in the early endosomes. After acidification of the endosome and subsequent membrane fusion, the genomic RNA segments of the influenza virus are released into the cytosol. The two major PRRs that are responsible for the cytoplasmic sensing of influenza virus infection are retinoic acid inducible gene-I (RIG-I) and NOD-like receptor family pyrin domain containing 3 (NLRP3) protein. Activation of RIG-I—by interaction with 5′ triphosphorylated RNA (5′ ppp-RNA)—results in the production of proinflammatory cytokines and type I interferons (IFNs), which in turn induce the expression of interferon-stimulated genes (ISGs). NLRP3 is a part of the NLRP3 inflammasome, which becomes activated by the proton gating activity of influenza A virus M2. This leads to the conversion of pro-IL-1β to IL-1β, which is a proinflammatory cytokine that is involved in the induction of Th17 cells and the expansion of CD4^+^ T cells.

The second barrier consists of proteins that bind to specific carbohydrate structures, so-called lectins. In the lung, the two main lectins involved in anti-influenza activity are surfactant proteins A (SP-A) and D (SP-D). These lectins hamper influenza virus infection by different mechanisms. SP-A is sialylated and therefore acts as a decoy receptor for influenza virus (γ-inhibition) [26], while SP-D binds mannose-rich oligosaccharides on influenza virus HA and NA proteins (β-inhibition)(Figure 1) [27].

#### 2.1.2. Sensing of Influenza Virus Infection

Once influenza virions have crossed the mucin- and lectin-rich layer that lines the respiratory tract, they reach respiratory epithelial cells. After recognition of the sialic acid-containing host cell receptors by the HA glycoprotein, endocytosis of the influenza virus is triggered and the virion particle ends up in the early endosomes. The passage in the endosomes allows entry of protons and at a later stage potassium ions into the virions which primes them for genome delivery. Matrix protein 2 (M2) fulfills an important function in this process [28]. The interior pH of the endosome becomes acidic, which induces a conformational change in the HA protein. This leads to the insertion of the fusion peptide of HA into the host membrane, and formation of a fusion pore. This pore allows the release of the genomic RNA segments of the influenza virus into the cytosol [29].

The two major pattern recognition receptors (PRRs) that are responsible for the cytoplasmic sensing of influenza virus infection are retinoic acid inducible gene-I (RIG-I) and NOD-like receptor family pyrin domain containing 3 (NLRP3) protein (Figure 1) [30,31]. Activation of RIG-I by interaction with 5′ triphosphorylated RNA, results in the production of proinflammatory cytokines and type I interferons (IFNs), which in turn induce the expression of interferon-stimulated genes (ISGs) via the JAK/STAT signaling pathway [32]. One of the most important antiviral ISGs is the *myxovirus* (MX) gene, which encodes the MxA and MX1 protein in human and mouse, respectively. Mx proteins are dynamin-like GTPases with strong and broad antiviral activity [33]. Type I IFNs also modulate the adaptive immune response: they form a bridge between innate and adaptive immunity by stimulating dendritic cells (DCs), which results in enhanced antigen presentation to CD4^+^ and CD8^+^ T cells [34]. NLRP3 is a part of the NLRP3 inflammasome, which becomes activated by the proton gating activity of influenza A virus M2 [35]. This leads to the activation of caspase-1 and conversion of pro-IL-1β to IL-1β, which is a proinflammatory cytokine that is involved in the induction of T helper 17 cells and the expansion of CD4^+^ T cells [36,37].

#### 2.1.3. Alveolar Macrophages

**Figure 2:**
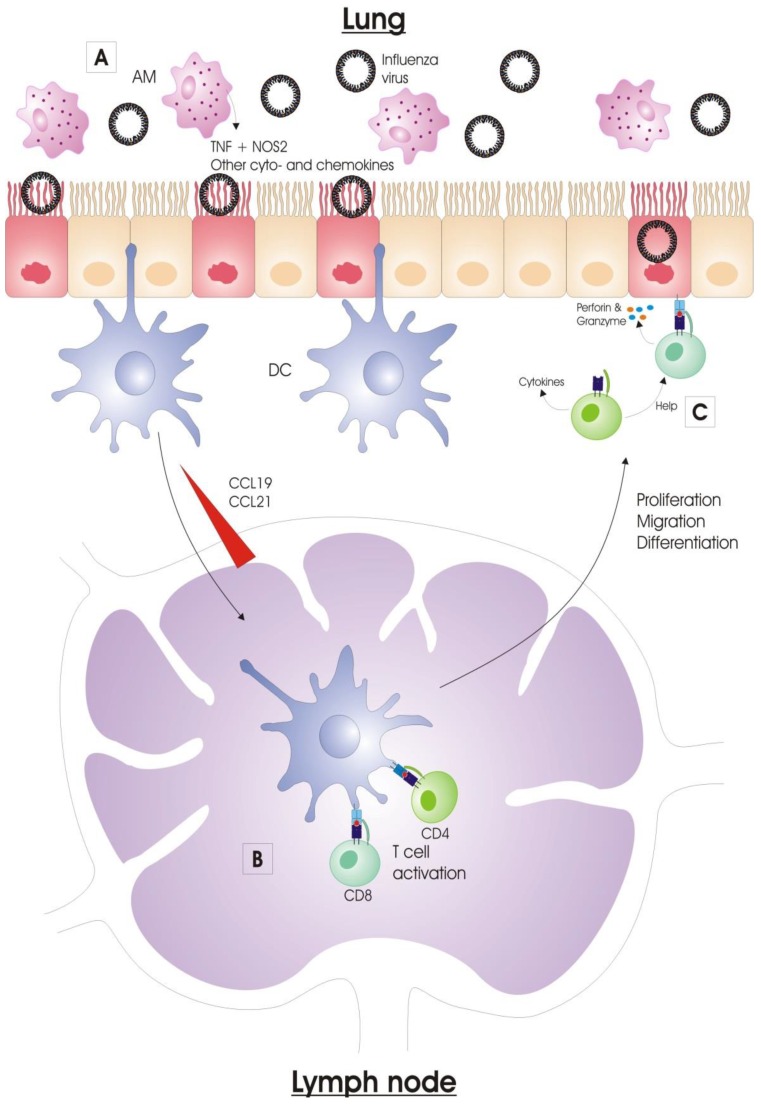
Adaptive immunity against influenza virus infection. (**A**) Alveolar macrophages (AMs) reside in the alveolar lumen and are considered the first immune cell type to encounter respiratory pathogens. Activated AMs produce nitric oxide synthase 2 (NOS2) and tumor necrosis factor (TNF), by which they also contribute to lung pathology upon influenza virus infection; (**B**) Dendritic cells (DCs) are professional antigen-presenting cells (APCs). The conventional DCs (cDCs), which are located underneath the airway epithelium barrier and above the basal membrane, continuously monitor the airway lumen with their dendrites. Once the cDCs acquired viral antigen, they migrate to the draining lymph nodes (DLNs) via a CCL19 and CCL21 chemokine gradient along the afferent lymphatic system. In the LNs, DCs present processed antigen to naïve T cells. The DC degrades viral proteins, and the resulting peptides (antigens) are presented by MHC class I or II proteins for specific CD8^+^ and CD4^+^ T cells respectively. The T cells that are specific for the antigen become activated and expand clonally. The newly activated T cells begin to acquire effector functions, and migrate from the LNs to the site of infection; (**C**) CD8^+^ T cells can kill infected respiratory epithelial cells, and, by this, clear the virus from the lungs. This is done by exocytosis of granules that contain perforins and granzymes. CD4^+^ T cells on the other hand mainly provide help to other immune cells, and regulate the immune response by producing a vast array of cytokines.

Alveolar macrophages (AMs) reside in the alveolar lumen and are considered the first immune cell type to encounter respiratory pathogens. When activated, AMs phagocytose virus particles and (apoptotic) infected cells, and consequently limit viral spread [38,39]. Activated alveolar macrophages also produce nitric oxide synthase 2 (NOS2) and tumor necrosis factor (TNF), by which they also contribute to lung pathology upon influenza virus infection (Figure 2) [40,41]. In addition, AMs regulate the adaptive immune responses. Depletion of AMs prior to influenza virus infection increases the primary cytotoxic T cell response in mice [42]. In pigs, conversely, depletion of AMs prior to influenza virus infection reduces the antibody titers and the number of virus-specific cytotoxic T cells. This effect was probably caused by an alteration of the expression pattern of inflammatory cytokines, since pro-inflammatory cytokines TNF and IFNγ are downregulated in the lung in contrast to the anti-inflammatory cytokine IL-10, which is upregulated in these animals after AM-depletion and subsequent influenza virus infection [38]. The downregulation of pro-inflammatory cytokines is also observed in mice infected with a reassorted H1N1 virus containing the 1918 Spanish flu HA and NA after AM-depletion [39]. AMs are very important for certain adaptive immune responses against influenza viruses. For instance, our group previously showed that AMs are essential for protection by anti-M2e IgG [43].

#### 2.1.4. Dendritic Cells

Dendritic cells (DCs) are professional antigen-presenting cells (APCs) which form an important link between the innate and adaptive immune systems. DCs can be considered the sentinels of the vertebrate immune system at body surfaces. In the lung, DCs perform multiple tasks, such as recognition and acquisition of antigens derived from pathogens and allergens, antigen transportation to the draining lymph nodes and induction of CD4^+^ and CD8^+^ T cell immunity [44]. The conventional DCs (cDCs) are located underneath the airway epithelium barrier and above the basal membrane. They continuously monitor the airway lumen with their dendrites, which they can extend into the airway lumen through the tight junctions of the epithelial cell layer [45]. During an infection, DCs will initiate the adaptive immune response by presenting viral antigens to B and T lymphocytes in the draining lymph nodes (DLNs) [46,47].

#### 2.1.5. Natural Killer Cells

Natural killer cells (NKs) are cytotoxic effector cells of the innate immune system. They can lyse influenza virus-infected cells following direct or indirect recognition of such target cells. The natural propensity of the cytotoxicity receptors NKp44 and NKp46 to recognize HA on the surface of infected cells contributes to a direct elimination of such cells [48,49]. Indirect recognition and subsequent lysis of infected cells by NKs is mediated by immune complexes at the surface of IgG-opsonized influenza virus-infected cells in a process called antibody-dependent cell cytotoxicity (ADCC) [50].

### 2.2. Adaptive Immunity

#### 2.2.1. Activation of Antigen-Presenting Cells

DCs can be considered the most important APCs in the lungs. In the steady state lung, several distinct DC subsets are present. These subsets differ in phenotype, anatomic location, and function. The CD103^+^ and the CD11b^hi^ DC subsets, located intraepithelial and submucosal/interstitial, respectively, are the main DC populations in the lungs and are collectively often referred to as cDCs [51].

After activation, respiratory cDCs have to acquire antigen derived from influenza virus for delivery to the DLN. The acquisition of antigen by DCs occurs via several mechanisms (Figure 2). The most likely way is by direct infection of the DCs, but different DC subsets differ in susceptibility to influenza virus infection [52]. Such an infection is probably abortive, so DCs fail to release infectious virions, but acquire sufficient amounts of viral antigens that are processed and uploaded into their MHC-I and -II molecules [53]. Another mechanism for cDCs to acquire viral antigen is by phagocytic engulfment of cell-free virions or dying/dead infected cells that harbor viral antigen. Lastly, antigen-acquisition can also occur through a membrane nibbling process, called trogocytosis [54,55,56].

#### 2.2.2. Antigen Presentation

Once the cDCs acquired viral antigen, they migrate to the draining lymph nodes via the afferent lymphatic system. This migration occurs along a CCL19 and CCL21 chemokine gradient, and is dependent on expression of the chemokine receptor CCR7 [57]. Although there are other APCs present in the DLN (such as plasmacytoid DCs, monocyte-derived DCs and macrophages), cDCs serve as the primary APCs for naïve influenza-specific T cells [58]. CD103^+^ DCs are the most potent APCs for the activation of cytotoxic T lymphocytes (CTLs) after influenza virus infection. These DCs can engulf influenza virus infected cells, and process and present virus antigen from these cells to CTLs, a process that is known as cross-presentation. CD103^+^ DCs acquire an antiviral state, which is induced by type I IFN signaling and characterized by the elevated mRNA-levels of IFN-inducible genes such as *ISG 15*, *OAS1a*, *Mx1*, *Ifitm1*, *Ifitm3* and *PKR* [59]. CD103^+^ DCs represent the first wave of migration and antigen presentation to CTLs. Afterwards, influenza virus antigen is replenished by blood-derived CD11b^hi^ DCs that migrate to DLNs from the lung [60,61]. This is thought to serve as a form of amplification loop for the generation of CTLs. Both CD103^+^ and CD11b^hi^ DCs also activate naïve CD4^+^ T cells with equal efficiency [62].

Naïve T cells circulate between blood and lymph nodes, where they remain for 12–24 h before returning to the blood and visiting the next lymph node. In the LNs, DCs present processed antigen to these naïve T cells. The DC degrades viral proteins, and the resulting peptides (antigens) are presented by MHC class I or II proteins. MHC class I/antigen complexes are presented on the cell membrane for recognition by specific CD8^+^ cytotoxic T cells. MHC class II presentation of the antigen on the cell membrane is recognized by CD4^+^ T helper (Th) cells. The T cells that are specific for the antigen—i.e., the T cells with a matching T cell receptor (TCR)—become activated and expand clonally. This expansion is extensive, as one naïve T cell can give rise to tens of thousands of progeny T cells [63]. The freshly activated T cells begin to acquire effector functions, such as the ability to produce effector cytokines. For activated CD4^+^ T cells, the cytokine environment is very important to differentiate into different effector Th cell types [64].

#### 2.2.3. Lymphocyte Migration to the Infected Lung

In order to migrate from the LNs to peripheral tissues, activated T cells change the expression profile of homing molecules. Mature naïve T cells express lymphoid homing receptors CD62L and CCR7, which are necessary for migration to secondary LNs [65,66]. Once these T cells are activated after a DC encounter, they migrate to the site of infection. In order to get out of the LNs, downregulation of CD62L and CCR7, and upregulation of other receptors is necessary. It has already been shown that different T cell subsets express their own specific chemokine receptor repertoire after activation, which allows them to be recruited to different peripheral tissues [67]. Recruitment of activated T cells to the infected lung occurs via nonspecific and specific routes. CD11a, which is a subunit of the integrin lymphocyte function-associated antigen-1 (LFA-1), is responsible for the nonspecific recruitment of activated T cells into the lungs, because this protein is upregulated in activated T cells, and its ligand ICAM-1 (Intercellular adhesion molecule-1) is expressed in peripheral tissues [68]. Specific recruitment of activated T cells is more complicated and less well understood. Mikhak and colleagues observed that lung DCs are responsible for the upregulation of chemokine receptor CCR4 on effector T cells, which allows for selective recruitment into the infected lung, where CCL2, CCL3, CCL5, CCL17 and CCL22, the ligands of CCR4, are produced [69]. However, it seems that this is not the case for CD8^+^ T cells, or at least that other mechanisms cannot be excluded. For this T cell population multiple recruitment mechanisms are implied to get the cells to the lung interstitium. Galkina and colleagues showed that migration of effector CD8^+^ T cells is promoted by expression of the chemokine CCL5 in the lung interstitium [70]. Slütter et al. on the other hand, have indicated that expression of CXCR3 on antigen-specific memory CD8^+^ T cells, from vaccinated mice, is critical for their migration to the airways [71]. Then again, Lim and coworkers recently reported the importance of the chemokine CXCL12 which is mainly produced by neutrophils, for virus-specific recruitment of CD8^+^ T cells and antiviral effector functions [72].

#### 2.2.4. Viral Clearance

Once influenza-specific effector T cells have entered the respiratory tract, there is a significant impact on viral titers through the expression of cytokines (IFNγ, TNF, IL-4 and IL-10) and direct lysis of infected cells. CD8^+^ cytotoxic T cells mainly contribute to viral clearance through the cytotoxic elimination of influenza virus-infected respiratory epithelial cells. Such elimination is possible by two mechanisms, i.e., the release of perforins and granzymes by the activated T cell or the engagement of tumor necrosis factor (TNF) family members on the surface of target cells with their ligands. Both mechanisms result in apoptosis of the target cell [73]. Influenza-specific CD4^+^ Th cells act directly and indirectly on the viral clearance process. Primarily they act indirectly by producing cytokines and helping B cells and CD8^+^ T cells [74,75,76]. Th cells can also directly eliminate infected cells, but this mechanism of action is rather accessory to their function as helper cells [77].

## 3. T Cell Response to Influenza Virus Infection

### 3.1. Primary T Cell Response: Deflowering the T Cells

Once infected with influenza virus, the epithelial cells start producing inflammatory cytokines. The first cytokines are IFNα, TNF, IL-1α and IL-1β, followed by IL-6, IL-8, monocyte chemoattractant proteins (MCPs), and macrophage inflammatory proteins (MIPs). Several of these cytokines have a chemotactic function, and thus attract innate immune effectors and antigen presenting cells to the site of infection. DCs that got recruited this way, take up viral particles and influenza-derived antigens. This triggers DC activation, maturation and migration to the draining LNs.

#### 3.1.1. CD4^+^ T Cell Primary Responses

Once naïve CD4^+^ T cells have matured, they traffic from the thymus to the secondary lymphoid tissues. Here the influenza-specific CD4^+^ T cells interact with mature DCs who carry viral antigens, and get activated. The activated T cells start proliferating and acquire effector functions. Activated CD4^+^ T cells differentiate to different subsets (Th1, Th2, Th17, Tfh, Treg), which can be characterized by their own distinct cytokine pattern. This differentiation is primarily promoted by the cytokine environment in which they are formed [78]. T helper (Th) 1 cells mainly produce IFNγ, TNF and IL-2. In contrast, Th2 cells do not produce IFNγ, but they large amounts of IL-4, IL-5 and IL-13. The main function of Th1 cells is enhancing the pro-inflammatory cellular immunity [79,80], while Th2 cells promote non-inflammatory immune responses and can induce the production of most classes of antibodies, mainly IgG1 and IgE [81]. Influenza virus infection generates both a Th1 response, where CD4^+^ T cells produce IFNγ, TNF and IL-2 [75,82], as well as a Th2 response, which helps formation of a robust antibody response [83]. However, following influenza virus infection, the CD4^+^ T cell response is biased toward a Th1 effector response [84].

Next to Th1 and Th2 cells, there is a third major CD4^+^ T cell subpopulation called Th17 cells [85]. These cells are characterized by the production of IL-17 and IL-22 [86]. They play an important role in the protection against opportunistic bacterial pathogens, such as is seen after influenza virus infection. Influenza A virus, however, employs mechanisms that inhibit a strong Th17 response, leading to an increased susceptibility to secondary bacterial infections [87]. The direct role of Th17 cells in the immune response against influenza virus is not totally clear yet, with studies pointing to both a possible beneficial or detrimental effect of a Th17 response. On the one hand, it has been shown that IL-17 can protect mice against a lethal infection with influenza A/Puerto Rico/8/34 (H1N1) and A/Alaska/6/77 (H3N2), and that it has a critical role in recruiting B cells to the pulmonary site of infection. Additionally, the IL-17 response contributes to a better survival percentage after a lethal influenza A/Puerto Rico/8/34 infection [88,89]. However, other studies indicate that IL-17 and the Th17 response may contribute to severe pulmonary immunopathology after influenza virus infection [85,90], and pre-induction of the Th17 immune response results in an exacerbation of lung pathology after influenza virus infection [91]. However, only the latter study by Gopal and coworkers investigated the direct in vivo effect of Th17 cells on lung pathology in an influenza virus infection model. The two former studies investigate the effect of IL-17 during influenza virus infection, but use a different infection model [89,90]. The remaining studies make use of in vitro differentiated CD4^+^ and/or CD8^+^ T cells which produce IL-17, but cannot be compared because of the difference in research question and models used [85,88].

A fourth CD4^+^ T cell subset are the regulatory T cells (Tregs), which contribute to homeostasis of the immune system and tolerance to self-antigens. This subset on itself can be further subdivided into two groups, natural Tregs (nTregs) that are generated in the thymus by MHCII-dependent T cell receptor (TCR) interactions [92], and induced Tregs (iTregs) which are generated in the periphery during an immune response [93]. Influenza virus infection generally triggers a robust Treg response, where iTregs suppress antigen-specific CD4^+^ and CD8^+^ T cell proliferation and cytokine production in an antigen-dependent matter [94].

The last CD4^+^ T cell subset discussed in this review is made up by the follicular helper T cells (Tfh). These cells are paramount in the formation and function of germinal centers, which are located in secondary lymphoid organs, and are the primary sites of B cell affinity maturation [95]. Here, the Tfhs play a pivotal role in providing help signals to B cells, which are essential for their survival and proliferation. After somatic hypermutation, the highest affinity B cells are selected by Tfhs for another round of proliferation and mutation [96,97]. Most human vaccines work on basis of long term protective antibody responses, so Tfhs are probably mediators of development of protective immunity by vaccines. In fact, it has already been shown that Tfhs are a limiting factor for generating antibody responses after immunization [98]. Learning more about this CD4^+^ T cell subset might be essential for the further improvement of human vaccination.

#### 3.1.2. CD8^+^ T Cell Primary Responses

Naïve CD8^+^ T cells get activated in the draining lymph nodes after recognition of a viral epitope-MHCI complex on an antigen presenting cell. This interaction initiates the differentiation of naïve CD8^+^ T cells into mature CTLs. CTLs then migrate to the site of infection, which in the case of an influenza virus infection is the infected lung [99]. There, their main task is killing infected respiratory epithelial cells, and, by this, clearing the virus from the lungs. To kill infected cells, CTLs use two distinct cytotoxic mechanisms: (1) granule exocytosis; and (2) the engagement of tumor necrosis factor (TNF) family members with their respective ligands. Granule exocytosis refers to the lysosomes that are released by CTLs after they interacted with influenza virus antigen-derived peptides complexed with MHCI on the infected cell through their T cell receptor (TCR). These granules contain the pore-forming protein perforin and serine proteases called granzymes. Perforin forms pores in the target cell membrane, which allows the pro-apoptotic granzymes to enter the cells and initiate programmed cell death. Cytotoxicity can also be mediated by several members of the TNF family, with FasL being the most important example of these family members. TCR engagement of peptide/MHCI-complexes results in upregulation of FasL and its migration to the cell membrane of CTLs. Fas proteins are expressed on the cell membranes of most cell types, and these proteins get oligomerized by interacting with their ligand FasL. This oligomerization triggers an apoptotic cascade in the target cell, resulting in programmed cell death [100]. Another cytotoxicity-inducing TNF family member is the TNF-related apoptosis-inducing ligand (TRAIL). TRAIL is comparable to FasL, in that interaction with its ligand (TRAIL-death receptor) also initiates an apoptotic pathway in the target cell. Evidence for its role in protection against influenza virus was reported by Brincks et al. who showed that TRAIL-deficiency decreases CD8^+^ T cell-mediated cytotoxicity, leading to more severe influenza virus infections [101].

### 3.2. Memory T Cell Response: The T Cells Remember

After clearance of the primary infection in the lungs, the effector T cells go into a contraction phase. Herein, 90%–95% of all antigen-specific T cells undergo apoptosis. The small portion of cells that remains are destined to be long-living memory T cells [102,103]. How this transition takes place, and how the cell-fate of a T cell is decided, is still unclear. Several models have been proposed and are reviewed by Buchholz et al. [104]. What is known for sure, is that the formation and homeostasis of memory CD4^+^ and CD8^+^ T cells is dependent on IL-7 and IL-15 [105,106,107,108,109]. Upon reinfection by the same pathogen, memory T cells begin producing effector molecules, undergo a massive clonal expansion, and differentiate to secondary effector T cells in order to control the infection faster. Some key features of memory T cells—which makes them able to respond faster than ‘normal’ effector T cells—are a high proliferative potential, a multipotent state, long term survival and self-renewal in absence of antigen (in presence of IL-7 and IL-15) [110].

The past fifteen years memory T cells have been subject of many studies, which led to the current ”classification” of memory T cells. For a long time it was thought that memory T cells could be divided into two groups, the high CCR7- and CD62L-expressing central memory T cells (Tcm) and low CCR7- and CD62L-expressing effector memory T cells (Tem). The first group of memory T cells patrols secondary lymphoid organs like naïve T cells do, but upon antigen-recognition they undergo a rapid and robust proliferation, differentiation and migration to the site of infection. The latter group recirculates between blood and non-lymphoid tissues [111]. Upon antigen-recognition they rapidly execute their effector functions like freshly stimulated effector T cells would do [111]. More recently, a third memory population was defined: tissue-resident memory T cells (Trm), which are derived from precursors that entered the tissue during the immune effector phase and remained in this tissue [112,113]. Instead of CCR7 and CD62L, Trm-specific markers are the glycoprotein CD69 and the integrin CD103 [114,115,116], although the expression of the latter is more predominant on CD8^+^ Trm cells than on CD4^+^ Trm cells [112]. CD4^+^ Trm cells are better associated with expression of CD69 and CD11a [113,117].

#### 3.2.1. CD4^+^ Memory T Cells

CD4^+^ memory T cells have long been studied less intensely than CD8^+^ memory T cells. The main reason for this is that CD4^+^ memory T cells do not expand as exuberantly as CD8^+^ memory T cells, and consequently are not present in large numbers after re-exposure to antigen [118]. For respiratory viruses, CD4^+^ Trm cells seem to be important for optimal protection against reinfection [113,119]. For influenza virus, it has been shown that CD4^+^ T cell epitopes are conserved within different subtypes of influenza virus. Interestingly, in people infected with seasonal influenza virus, virus-specific CD4^+^ T cells have been isolated which cross-react with emerging reassortant strains like H5N1 [120]. Alexander and colleagues have shown that DNA vaccines, containing several CD4^+^ T cell epitopes, protected against lethal influenza virus infection [121]. Next to the circumstantial evidence, recent studies have elucidated a lot more about the role of CD4^+^ memory T cells in the immune response to influenza virus infection. It has been shown that CD4^+^ memory T cells can direct influenza virus clearance in absence of B cells and CD8^+^ T cells, however they cannot provide full protection [122]. In cooperation with other cell types, however, CD4^+^ memory T cells can provide clear protection during re-infection [123,124,125]. These findings have great significance for the production of universal influenza vaccines that aim at inducing long lived T cell responses.

#### 3.2.2. CD8^+^ Memory T Cells

Memory CD8^+^ T cells, like CD4^+^ memory T cells, have the ability to rapidly generate effector functions. They also produce a burst of secondary CTLs that can rapidly contain secondary infections. Repetitive reactivation of the memory CD8^+^ T cells, either through booster vaccinations or successive infections, augments the effector-like properties of memory CD8^+^ T cells and the frequency of Tem cells in the resulting memory T cell pool [126]. The importance of memory CD8^+^ T cells has already been illustrated in humans. People without detectable pre-existing antibodies to the 2009 pandemic H1N1 strain were monitored following the global spread of this virus. From this it was evident that people that showed no or minor disease symptoms had higher levels of pre-existing IAV-specific CD8^+^ Tem cells. This study also showed no clear correlation between disease severity and pre-existing memory CD4^+^ T cells [127]. This is rather striking because Wilkinson and colleagues noticed an inverse correlation between the presence of pre-existing CD4^+^ memory T cells and disease severity following a controlled challenge [128]. The reason for these different observations is currently unclear.

CD8^+^ Trm cells form the frontline against secondary infection by influenza viruses in the lung compartment. Upon reinfection, they can immediately acquire effector functions. How come that these CD8^+^ Trm cells are not infected by influenza viruses, which, after all, merely require a sialic acid-containing receptor on a mammalian cell for their entry? It has been reported that CD8^+^ Trm cells that are formed after an influenza virus infection, show a massive upregulation of the interferon-induced transmembrane protein 3 (IFITM3) [129]. The same observation was made in CD8^+^ Trm cells from the brain after infection with Vesicular Stomatitis Virus [130]. IFITM3 is a potent antiviral molecule that can render resistance to infection with influenza virus by interfering with the fusion of the influenza virus envelope and the late endosome cell membrane, more specifically by blocking the formation of fusion pores [131]. All these observations make it very clear that generating memory T cells should be one of the most important objectives in the development of potent anti-influenza vaccines.

## 4. Vaccines

### 4.1. Current Influenza Vaccines

Annual influenza vaccination is the most effective way to prevent influenza virus infection [12], and is generally done with inactivated (TIV or QIV) or live-attenuated vaccines (LAIV). TIV induces higher titers of serum hemaglutination-inhibiting (HI) IgG and IgA antibodies than LAIV [132]. LAIV in its turn induces higher levels of nasal-wash IgA than TIV which mainly elicits IgG in the upper respiratory tract mucosa [133,134]. Inducing efficient cellular immune responses in the lung is dependent on efficient replication of the virus in the lung. TIV does not elicit cellular immunity because it is inactivated, and LAIV is designed in such a way that it only replicates in the upper respiratory tract. Therefore, both vaccines do not show much potential for inducing efficient cellular immune responses. However, cellular immunity was demonstrated in the lung after LAIV-administration in animal models [135]. Additionally, in humans, there is also an indication that LAIV elicits cellular immunity [21].

In the past, LAIV seemed to be superior to TIV in reducing laboratory-confirmed influenza in children [136]. Consequently, the use of LAIV was recommended in the UK, Germany, Israel, Canada and Sweden for children of various ages [137], and in the USA for children aged two to eight years [138]. In adults, the efficacy of LAIV is somewhat debated. In a study conducted by Monto and colleagues, LAIV was 50% less efficient than TIV in the reduction of laboratory-confirmed influenza during the 2008–2009 influenza season [139]. Nichol and colleagues showed less efficacy in subjects between the age of 50 and 64, which led to the disapproval of LAIV for this age group in the USA [140]. Other studies show that LAIV reduces illness by 85% compared to TIV (subjects aged 18–41 years), and that LAIV does protect against influenza virus infection in adults above the age of 60 years [132,141]. However, as mentioned earlier, use of LAIV is no longer recommended for the upcoming 2016–2017 season because of its poor effectiveness against the H1N1 pandemic of 2009 [16,17].

### 4.2. T Cells in Heterologous Protection against Influenza Viruses

Current vaccines are mainly designed to elicit humoral rather than cellular immunity. The protective cut-off for evaluating vaccines is hence based on a hemaglutination-inhibition (HI) titer of 1/40 or more. This cut-off already dates from 1972, when Hobson and colleagues showed that a HI-titer of 1/36 corresponded with a 50% reduction of infection rate [142]. However, the 1/40 HI-titer correlate of protection is not a good cut-off for evaluating the vaccine-efficacy in children and elderly people [143,144]. Alternatively, a new correlate of infection should be developed, which takes into account the HI-titer and the influenza-specific cellular immune response. A vast amount of epidemiological studies supports this statement. During the 1977 influenza season, protection against homologous H1N1 virus infection was illustrated when H1N1 re-emerged after 20 years. Individuals born before 1955 had a lower influenza A/H1N1 attack rate in 1977 than people born after 1955 [145]. A surveillance study performed during more than 10 years in families in Cleveland showed that adults who were repeatedly infected with influenza virus before the pandemic year 1957 (H2N2), had a much lower incidence of influenza during the pandemic year [146]. Re-analysis of this study provided indirect proof that cellular immunity is involved in long-lived protection against influenza virus infection [147]. Studies performed during and after the 2009 Mexican flu pandemic showed comparable results [127,148]. In an experimental challenge study performed by McMichael et al., a direct correlation between pre-infection levels of CTLs and virus clearance was detected [149]. Similarly, in another controlled infection experiment, a strong protective role for memory CD4^+^ T cells was elucidated [128]. These studies were the starting point for the mounting evidence of the role of T cells in protection against influenza virus.

### 4.3. Vaccination-Induced T Cell Immunity: To Serve and Protect

People that got infected with influenza virus in the past, have a two-part defense to neutralize a homologous reinfection. The first line of defense consists of antigen-specific antibodies that were elicited by the previous influenza virus infection. This is considered as neutralizing immunity, because the antibodies can directly attack the virus particles. However, often not all of the inoculum is neutralized or opsonized by the antibodies, which leaves some viral particles able to infect host cells. This is where the cellular immune response also kicks in. Vaccination strategies should induce both lines of the defense mechanism, and ideally in such a way that there is broad heterologous protection against subsequent influenza virus infections. As mentioned before, current inactivated vaccines do not elicit a strong T cell response. However, this might, in part, be overcome by the use of the right adjuvants. Adjuvants help to determine the type and size of the immune response provoked by vaccination. They can act as a depot and stabilizer for the vaccine, and stimulate innate immune responses, affecting the subsequent adaptive immune responses. The only adjuvants approved for use in humans are aluminum salts (aluminum hydroxide, aluminum phosphate and aluminum sulphate) and squalene-based adjuvants (AS03 and MF59); these adjuvants have been shown to promote induction of T cell responses [150]. Inactivated vaccines elicit neutralizing antibodies, which could impair the induction of cross-reactive cellular responses. This is of special concern for young children who are in general immunologically naïve for influenza virus [22]. This problem can be avoided by inducing infection-permissive immunity, which may still be protective, but allows virus-induced cross-reactive immune responses. A vaccine which targets the conserved ectodomain of influenza virus matrix protein 2 (M2e) delivers this kind of non-neutralizing immunity, since anti-M2e antibodies rely on Fc receptors and innate immune components to provide protection [43]. Another promising vaccine candidate targets the influenza virus neuraminidase (NA) glycoprotein. Antibodies that inhibit NA-activity do not block entry of the virus into the host cell, so such antibodies do not provide sterilizing immunity and may contribute to immunity against a virus that has a similar NA type [151].

Last years, recombinant T cell-inducing vaccines are getting into play, the most advanced vaccine candidate is based on Modified Vaccinia Ankara (MVA) viruses expressing influenza virus NP and M1 antigens [152]. Individuals vaccinated with this MVA−NP+M1 show an increase in IFNγ-producing (cytotoxic) CD8^+^ T cells, and consequently an increase in protection against influenza virus infection [153,154]. Coadministration of this MVA-based vaccine with the seasonal TIV results in an increase of influenza strain-specific antibody responses and a boost of memory T cells capable of recognizing a range of influenza A subtypes [155].

FLU-v is a new vaccine candidate which consists of four peptides (three peptides derived from influenza A virus (IAV), and one peptide from influenza B virus (IBV)). These peptides are synthetic multi-epitope peptides that were identified in silico [156]. The vaccine is already proven to be safe and well-tolerated in phase I clinical trials [157]. Peripheral blood mononuclear cells (PBMCs) from vaccinated individuals showed more IFNγ-production in comparison to those of non-vaccinated individuals, but this did not lead to a decrease of symptoms after challenge [158].

The most recently reported T cell inducing vaccine candidates are FP-0.01 (Flunisyn^TM^) and Multimeric-001 developed by Immune Targeting Systems Limited (London, UK) and BiondVax Pharmaceuticals Ltd. (Ness Ziona, Israel) respectively. The first vaccine consists of six peptides derived from IAV NP, M1, PB1 and PB2 proteins. These peptides are linked to fluorocarbon, which self-assembles into micelles omitting the need for adjuvants and increasing the peptides’ in vivo half-life [159]. After vaccination of healthy volunteers with FP-0.01, the frequency of IFNγ-producing CD8^+^ T cells was only slightly increased. However, PBMCs isolated from these vaccinated individuals showed an increased IFNγ-production, in comparison to unvaccinated individuals, following stimulation. Multimeric-001, is composed of a large protein comprising nine highly conserved peptides derived from IAV HA, NP and M1 proteins. PBMCs from vaccinated individuals show an increased proliferation and cytokine-production in comparison to PBMCs from non-vaccinated individuals after stimulation with peptides derived from influenza virus NP and HA proteins [160].

## 5. Conclusions

In order to elicit robust, long lasting, heterosubtypic immunity, there are some considerations to keep in mind when developing new vaccine candidates. Cross-reactive CD8^+^ CTL-mediated immunity plays an important part in controlling IAV infections, so it would be logical to focus on inducing a potent memory CD8^+^ T cell response. To induce such a strong CD8^+^ T cell response, efficient antigen-presentation by APCs is important. For this, it is interesting to target relevant DC subsets—such as CD8^+^ and CD103^+^ DCs—for maximal IAV-specific CTL-stimulation [161]. The important role of DCs to activate CD8^+^ T cells makes them attractive targets for vaccination. One approach to develop such so-called DC vaccines is by targeting antigen to PRR-activated APCs in an antibody-dependent way to achieve a strong initial T cell response [162]. Studies in animal models have demonstrated that targeting protein vaccines to lung dendritic cells even leads to the generation of Trm CD8^+^ T cells protective against IAV challenge [163,164]. In humans however, little is known about the protective potential of lung Trm cells, but they are important to consider when designing a novel vaccine candidate. To generate resident memory T cells by means of vaccination, one has to keep in mind that the number of generated memory T cells is determined by the size of the burst phase of the initial T cell response. Therefore, it is essential to induce an effector T cell population that is as large as possible. Recruiting antigen-specific CD8^+^ T cells is dependent on the presentation of antigen to the T cells, so the amount of presented antigen is crucial for the Trm cell population size [163]. In addition, the antigen-presentation at later time points is essential for generating a good Trm response. To achieve this, booster vaccines given at the correct moment might skew the Trm cell populations to higher affinity clones. A recent study showed that rapid boosting after vaccination with LAIV generated superior CD8^+^ T cell memory [165]. Research suggests that these boosters can give rise to a robust memory T cell response as well as enhance Tfh responses, because these cells are also dependent on late antigen-encounters [166,167]. Taking the memory T cells into account, this kind of vaccination strategies will generate a better antibody response and a more robust cellular response which is cross-reactive to new influenza strains.

## Abbreviations

The following abbreviations are used in this manuscript:

ADCC	Antibody-dependent cellular cytotoxicity
AM	Alveolar macrophage
APC	Antigen-presenting cell
CCL	Chemokine (C-C motif) ligand
CCR	C-C chemokine receptor
CD	Cluster of differentiation
CD62L	L-selectin
Cdc	Classical dendritic cell
CTL	Cytotoxic T lymphocyte
CXCL	Chemokine (C-X-C motif) ligand
DC	Dendritic cell
DLN	Draining lymph node
DNA	Deoxyribonucleic acid
FasL	Fas ligand
GTP	Guanosine triphosphate
HA	Hemagglutinin
HI	Hemagglutination-inhibiting
IAV	Influenza A virus
ICAM-1	Intercellular adhesion molecule 1
IFITM3	Interferon-induced transmembrane protein 3
IFN	Interferon
Ig	Immunoglobulin
IL	Interleukin
ISG	Interferon-stimulated gene
iTreg	Induced regulatory T cell
LAIV	Live-attenuated influenza vaccine
LFA-1	Lymphocyte function-associated antigen 1
M	Matrix protein
MCP	Monocyte chemoattractant protein
MHC	Major histocompatibility complex
MIP	Macrophage inflammatory protein
mRNA	Messenger ribonucleic acid
MVA	Modified Vaccinia Ankara
Mx	Myxovirus resistant
NA	Neuraminidase
NK	Natural killer
NLRP3	NOD-like receptor family pyrin domain containing 3 protein
NOS2	Nitric oxide synthase 2
NP	Nucleoprotein
nTreg	Natural regulatory T cell
PB	polybasic
PBMC	Peripheral blood mononuclear cell
PRR	Pattern recognition receptor
QIV	Quadrivalent inactivated influenza vaccine
RIG-I	Retinoic acid-inducible gene I
RNA	Ribonucleic acid
SP	Surfactant protein
Tcm	Central memory T cell
TCR	T cell receptor
Tem	Effector memory T cell
Tfh	Follicular helper T cell
Th	T helper cell
TIV	Trivalent inactivated influenza vaccine
TNF	Tumor necrosis factor
TRAIL	TNF-related apoptosis-inducing ligand
Treg	regulatory T cell
Trm	Resident memory T cell
WHO	World Health Organization
